# Understanding the neural bases of bodily self-consciousness: recent achievements and main challenges

**DOI:** 10.3389/fnint.2023.1145924

**Published:** 2023-06-19

**Authors:** Zoé Dary, Christophe Lopez

**Affiliations:** Aix Marseille Univ, CNRS, Laboratory of Cognitive Neuroscience (LNC), FR3C, Marseille, France

**Keywords:** consciousness, body representation, self-consciousness, multisensory integration, neuroimaging

## Abstract

The last two decades have seen a surge of interest in the mechanisms underpinning bodily self-consciousness (BSC). Studies showed that BSC relies on several bodily experiences (i.e., self-location, body ownership, agency, first-person perspective) and multisensory integration. The aim of this literature review is to summarize new insights and novel developments into the understanding of the neural bases of BSC, such as the contribution of the interoceptive signals to the neural mechanisms of BSC, and the overlap with the neural bases of conscious experience in general and of higher-level forms of self (i.e., the cognitive self). We also identify the main challenges and propose future perspectives that need to be conducted to progress into the understanding of the neural mechanisms of BSC. In particular, we point the lack of crosstalk and cross-fertilization between subdisciplines of integrative neuroscience to better understand BSC, especially the lack of research in animal models to decipher the neural networks and systems of neurotransmitters underpinning BSC. We highlight the need for more causal evidence that specific brain areas are instrumental in generating BSC and the need for studies tapping into interindividual differences in the phenomenal experience of BSC and their underlying mechanisms.

## 1. Introduction

The last decades have seen a surge of interest in the neural mechanisms underpinning self-consciousness. A very productive line of research has investigated the brain mechanisms of bodily self-consciousness (BSC), showing that they rely on several bodily experiences and multisensory integration. BSC encompasses bodily experiences such as *self-location*—the experience of occupying a volume of space, typically localized within the body; *body ownership*—the experience of owning a body; *agency*—the sense of being in control of one’s own movements; and *first-person perspective*—the experience of an egocentric perspective on the world and the self (Blanke, 2012).

Pioneer descriptions of patients with stroke or epilepsy associated with disturbances in one or several aspects of BSC suggested that parts of the cortex and thalamus were involved in the experience of being a self, of being embodied, of owning a body, or of having the control over actions and thoughts (e.g., Lhermitte, 1939; Gerstmann, 1942; Hécaen and de Ajuriaguerra, 1952). For example, seizures and lesions in the temporo-parietal junction have consistently been associated with illusory disembodied self-location (Blanke et al., 2004), and damage to the insula and parietal cortex have consistently been associated with disownership of body parts (Berlucchi and Aglioti, 2010).

Neuroscientists have more recently put BSC under scientific scrutiny by combining pluridisciplinary approaches from experimental psychology, virtual reality, neuroimaging, electrophysiology, physiology and robotics (Blanke et al., 2015; Bernasconi et al., 2022; Monti et al., 2022). A fruitful approach developed during the last 20 years to identify the neural bases of BSC has been to manipulate bodily experiences by creating multisensory illusions, and to explore the neural correlates of these changes in BSC. Two widely used and popular experimental paradigms in cognitive neuroscience were the “rubber hand illusion” (Botvinick and Cohen, 1998) and the “full-body illusion” (Ehrsson et al., 2004; Lenggenhager et al., 2007; Slater et al., 2009), developed to manipulate illusory self-identification with a fake hand or body, a mannequin, or a virtual hand or body. Non-invasive functional neuroimaging combined to these illusions revealed that BSC involves the premotor cortex, middle and inferior temporal cortex, extrastriate body area, inferior parietal sulcus, primary somatosensory cortex, insula, anterior cingulate cortex, and the temporo-parietal junction (e.g., Ehrsson et al., 2004; Tsakiris et al., 2007, 2008; Ionta et al., 2011b; Petkova et al., 2011).

As part of the Research Topic *Insights in Integrative Neuroscience 2022*, the present literature review aims at identifying novel developments, current challenges and future perspectives (see^1^ and Buhusi et al. (2023), in this Research Topic) in the analysis of the neural bases of BSC. We do not intend to be exhaustive in this non-systematic review of the literature, and present a selection of lines of research that, according to us, were particularly important in the field in that they contributed to the multilevel analysis of BSC (i.e., from neurochemistry to neurons, neural assembly, and whole brains, across species and across the lifespan). Relevant references were selected after searching in PubMed articles published in English in the last 10 years about BSC [and the main bodily experiences underpinning BSC: self-location, agency, first-person perspective, body ownership; see Blanke (2012)]. On the basis of this literature search, 60 articles were identified, among which we highlighted results from articles published in mostly the last 5 years, that provided novel developments into the understanding of the neural bases of BSC. We summarized results from studies providing a better understanding of the contribution of silent senses (e.g., interoceptive signals) to the neural mechanisms of BSC, results from studies investigating the overlap between the neural mechanisms of BSC and higher-level forms of self (i.e., the cognitive self), and novel approaches from structural and functional connectivity to decipher the neural network underpinning BSC. We also identify the main current challenges and propose future perspectives that need to be conducted to progress into the understanding of the neural mechanisms of BSC. In particular, we point the lack of crosstalk and cross-fertilization between subdisciplines of integrative neuroscience to better understand BSC, especially the lack of research in animal models to decipher the neural networks and systems of neurotransmitters underpinning BSC.

## 2. Recent developments and major accomplishments

### 2.1. Neural mechanisms of interoception contribute to BSC

Neuroscientific investigations of BSC have increasingly paid attention to the link between BSC and interoceptive signals, i.e., signals from receptors in inner organs and viscera (heart, stomach, blood vessels, kidneys…) contributing to homeostasis. Although interoceptive signals are often processed in a pre-reflective way (we are usually not aware of our heart beats, unless we are very excited or scared), they also contribute to a large range of higher-order functions, such as emotions and decision making (Craig, 2009; Khalsa et al., 2018). There is also evidence that interoceptive signals contribute to BSC (Aspell et al., 2013; Seth, 2013).

A line of research measured the participant’s ability to perceive their heartbeats [i.e., using heartbeat counting tasks or heartbeat tracking tasks (Garfinkel et al., 2015)] or their internal bodily states using questionnaires, and correlated these measures of interoceptive sensitivity to several bodily experiences related to BSC. For example, participants with higher interoceptive acuity tended to show lower illusory ownership, i.e., lower self-identification with a fake hand (Tsakiris et al., 2011), or tended to report higher anchoring of their self to their body (Nakul et al., 2020a).

**FIGURE 1:**
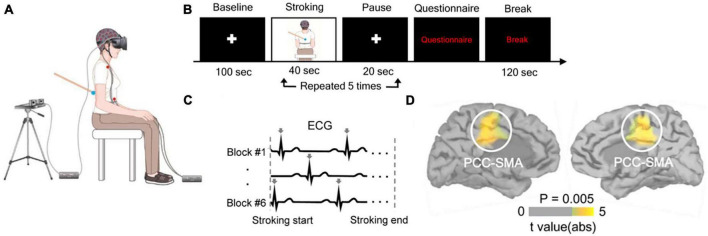
Heartbeat evoked potentials and bodily self-consciousness (BSC) in a full-body illusion. **(A)** Participants received on their back a tactile stimulation either synchronously or asynchronously with a video of their own back being stroked in a head-mounted display. **(B)** Participants answered a questionnaire about BSC (e.g., self-identification with the seen body) after five exposures to visuo-tactile stimulation. **(C)** Electrocardiogram was recorded during the visuo-tactile stimulation. Arrows indicate cardiac R-peaks. **(D)** Difference in activation by heartbeats between synchronous and asynchronous visuo-tactile stimulation was significant in the bilateral posterior cingulate cortex. Reproduced from Park et al. (2016).

Park et al. (2016) used electroencephalography and a method referred to as heartbeats evoked potentials (HEPs) to analyze how brain activity in response to heartbeats was modulated during experimentally-induced self-identification with a distant body (Figure 1). The authors demonstrated that HEPs amplitude decreased during illusory self-identification with a distant body, for electrodes located over frontocentral scalp regions. In addition, changes in BSC were correlated to HEPs amplitude. Source localization and cluster-based permutation tests indicated that the activity within the left and right posterior cingulate cortex, extending to the supplementary motor area, was associated with changes in BSC (i.e., self-identification with the avatar). The authors noted that using a less conservative statistical threshold, activity in the left insula, a crucial area for interoception, was also related to self-identification with an avatar. Using intracranial electroencephalography in eight patients with epilepsy, a technique characterized by a high spatiotemporal resolution, the same team confirmed that modulation of HEP amplitude during illusory self-identification with an avatar was related to neural activity in the insula (Park et al., 2018).

Altogether, data indicate that, in addition to exteroceptive visual and tactile signals, the brain processing of interoceptive signals contributes significantly to the neural bases of BSC [reviewed in Park and Blanke (2019)]. The data especially point to the importance of interoceptive information processing in the insula for BSC.

Another study analyzed the correlation between gut physiology and BSC in 31 healthy male participants (Monti et al., 2022). With a new method based on a wireless capsule ingested by participants, Monti et al. (2022) recorded temperature, pressure and pH across the entire gastrointestinal tract in real-time during a full-body illusion in virtual reality coupled with electrogastric rhythm recordings. The authors used questionnaires to measure illusory self-identification, self-location and agency over a virtual body breathing in synchrony or not with the participants. Results showed a relation between illusory self-location and pH: less acidic pH was associated with a stronger sense of occupying the same place as the virtual body. A lower feeling of disembodiment was also associated with higher gastrointestinal temperature. Electrogastrographic data indicated a relation between physiological activity of the stomach and self-identification with a virtual avatar.

The data reviewed above have strong implications in that they showed that BSC is directly related to objective physiological measures about the inner state of the body, in line with previous demonstrations of relations between illusory ownership over a fake body part and immune reaction (Barnsley et al., 2011). They open new avenues for clinical neuroscience to investigate whether conditions characterized by an abnormal sense of embodiment and sense of self (e.g., depersonalization) are related to abnormal brain processing of gut interoceptive signals.

### 2.2. Shared neural networks underpinning conscious experience of sensory events and BSC

**FIGURE 2:**
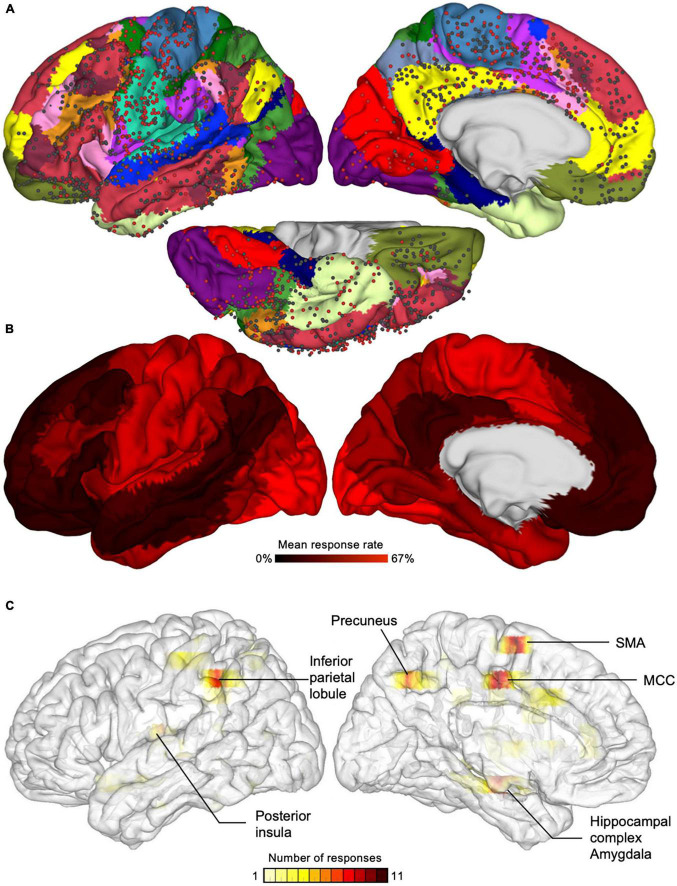
Responses elicited by electrical stimulation of the cortex in awake epileptic patients. **(A)** Circles represent the location of electrical stimulation applied in different epileptic patients. Red circles are sites where electrical stimulation evoked a change in the conscious experience or a motor response, whereas black circles are sites where no response was evoked. **(B)** The color code represents the mean response rate, showing high response rate in somato-motor and visual networks and low response rate in the default and limbic networks and in other transmodal networks. Reproduced from Raccah et al. (2021). **(C)** Color-coded density maps showing the number of electrical brain stimulation evoking disturbances of the bodily self in a systematic review of the literature, showing six main areas underlying the bodily self. MCC, middle cingulate cortex; SMA, supplementary motor area. Brain images have been flipped horizontally to allow comparisons with part **(B)**. Reproduced from Dary et al. (2023).

Cognitive neuroscientists and clinicians progressed in understanding the neural bases of consciousness by investigating *conscious experience* of sensory events (e.g., conscious perception of visual signals, sounds, touch), that is, “what it is like” to have a sensory experience (Nagel, 1974). An important question was to understand whether brain networks involved in the conscious experience of sensory events (without self-representation) overlap with those underpinning BSC (the experience of a bodily self). Invasive intracranial electrical stimulation in patients with epilepsy and during awake brain surgery have long shown that stimulation of various cortical and subcortical sites can evoke a variety of conscious experiences (Penfield, 1958; Halgren et al., 1978) devoid of self-representation (e.g., seeing phosphenes; auditory hallucinations: hearing human voice; somatosensory sensations: warmth, paresthesia, tingling, flexion of a finger; pain; vestibular responses: feeling of falling or flying; see Selimbeyoglu and Parvizi (2010), for a comprehensive review), or distortions in the sense of self (e.g., sensation of unreality, out-of-body experience, disownership; see Dary et al. (2023), for a comprehensive review). Fox et al. (2020) conducted an extensive whole-brain mapping of the effects of direct intracranial electrical stimulation in 67 patients with epilepsy (Figure 2A). The authors found that the elicitation rate of conscious experience (mainly devoid of self-representation) was high in rather unisensory parietotemporal and occipital cortices at the base of the cortical hierarchy. By contrast, they reported that electrical stimulation of the heteromodal cortices placed higher in cortical hierarchy, such as the frontal cortex, had a much lower elicitation rate (Figure 2B).

In a more focused investigation of the role of the prefrontal cortex based on the same data sample, Raccah et al. (2021) concluded that, with the exception of electrical stimulation of the orbitofrontal cortex and the anterior cingulate cortex, which can disturb conscious experience, electrical stimulation of the anterolateral prefrontal cortex does not seem to modify conscious “relation to the immediate environment.”

This result is important in that the prefrontal cortex has often been proposed as a crucial region for conscious experience, especially for partisans of the global workspace theory (Dehaene and Changeux, 2011; Boly et al., 2017; Odegaard et al., 2017). According to this theory, conscious experience “occurs when incoming information is made globally available to multiple brain systems through a network of neurons with long-range axons densely distributed in prefrontal, parieto-temporal, and cingulate cortices” (Dehaene and Changeux, 2011, p. 200). However, it appears that a conscious experience was mostly evoked or modified after stimulation of a large posterior network including the sensory cortices, most often not involving the prefrontal cortex (Koch, 2018; Raccah et al., 2021).

As introduced above, BSC is grounded on bodily experiences and the integration of sensory signals from the body and its immediate environment. Accordingly, neural activity within the multisensory and sensorimotor cortices is deemed crucial for the bodily self (Blanke, 2012). Figures 2B, C compares intracranial electrical brain stimulation evoking conscious experience not directly related to the self (Koch, 2018; Raccah et al., 2021) with stimulation evoking disturbances of the BSC, such as disembodiment, disownership of a body part, or abnormal sense of agency (Dary et al., 2023). A recent systematic review of the literature between 1937 and 2022 identified a total of 221 patients who reported altered BSC during electrical brain stimulation (Dary et al., 2023). Three-dimensional density maps of stimulation that most consistently altered BSC revealed the crucial contribution of the middle cingulum, inferior parietal lobule, supplementary motor area, posterior insula, hippocampal complex/amygdala, and precuneus (Figure 2C). Interestingly, these regions overlap with the cortices where the elicitation rate of conscious experience not directly related to the self was high (Figure 2B; Fox et al., 2020).

In conclusion, results from intracranial stimulation studies indicate a strong overlap between brain networks underpinning conscious experience of sensory events devoid of self-representation and those involved in BSC (disturbances of the bodily self). Thus, consciousness of a bodily self may share similar brain mechanisms with conscious experience, mostly grounded in the sensorimotor cortices, emphasizing again the embodied nature of BSC.

### 2.3. Shared neural networks underpinning the bodily self and the cognitive self

Self-consciousness is a multifaceted aspect of the mind: in addition to BSC (the bodily self), which is mostly pre-reflective and relies on multisensory and motor mechanisms (Blanke, 2012), higher-levels forms of self, sometimes referred to as “cognitive self” (or “narrative self”), are more reflective and involve a sense of self extended in time [Gallagher (2000); reviewed in Schaller et al. (2021)]. The cognitive self involves cognitive processes, such as self-recognition in a mirror, self-recognition of the own voice, autobiographical memory, language (e.g., the use of “I” and “me”), or mental time travel (Schaller et al., 2021). Whereas the bodily self and cognitive self are conceptually different, there are theoretical and experimental arguments to suggest that they cannot be separated (e.g., Zahavi, 2005), overlap partially, and that self-consciousness emerges from interactions between both levels (Gallagher, 2013). Damasio (1999), for example, distinguished a simple level of self (the “protoself,” related to the bodily self) from a more complex form of self (the “autobiographical self,” related to the cognitive self), which depends on cognition. According to Damasio (1999), the relation between these two levels of self is crucial, in that the autobiographical self relies on the foundation of the protoself. Phenomenal approaches of the self have also suggested that pre-reflective self-consciousness serves as a foundation for all reflective levels of the self (e.g., Legrand, 2007).

Given the conceptual overlap between the bodily and the cognitive self, recent studies endeavored to explore the extent to which BSC may share some mechanisms and neural substrates with high-level forms of self. A line of research investigated the contribution of brain networks supporting memory, as memory involves cognitive processes at the base of a self extended in time (i.e., a self with a past, a present, and a future). Bréchet et al. (2018) specifically tested whether BSC shares neural correlates with episodic and semantic aspects of autobiographical memory. The rationale was that “the subjective sense of self in time that enables us to re-experience ourselves in the past and mentally project ourselves into the future, i.e., autonoetic consciousness” (Bréchet et al., 2018; p. 2) (at the base of episodic autobiographical memory), involves core aspects of BSC, such as self-location and first-person perspective. Results from their meta-analysis of functional neuroimaging studies on autobiographical memory, compared to core areas of BSC identified by Ionta et al. (2011a) indicate an overlap between the regions involved in BSC and in episodic autobiographical memory in the bilateral angular gyrus. No overlap was found in other parietal and temporal areas. In addition, there was no overlap between BSC and semantic autobiographic memory. Thus, the angular gyrus appears to be a core region for multisensory processing related to the immediate bodily self experience (experience of perceiving the world from a first-person perspective and of being spatially located) and for later re-experiencing the self in episodic autobiographical memory.

### 2.4. Structural and functional connectivity to decipher the neural network underpinning BSC

In addition to the identification of the brain areas linked to changes in BSC, neuroimaging studies are now investigating the patterns of structural and functional connectivity which characterize the brain networks underpinning BSC. Rather than a pure localizationist approach as allowed by clinical case reports and case series, these more recent approaches allow predicting and decoding states of BSC as a function of the pattern of activity and connection within brain networks [reviewed in Thiebaut de Schotten and Forkel (2022)].

**FIGURE 3:**
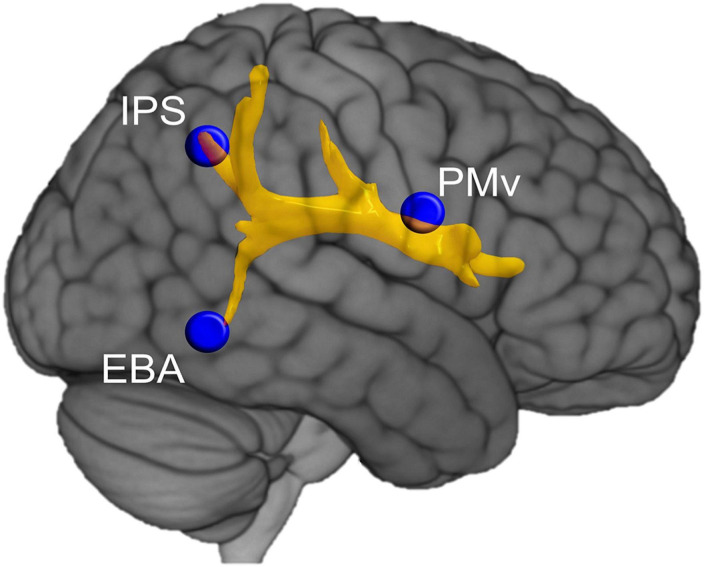
Structural connectivity underlying the sense of body ownership. Representation of the tracts damaged more frequently in stroke patients with disturbed sense of body part ownership when compared to patients whose sense of body part ownership was not affected. EBA, extrastriate body area; IPS, intraparietal sulcus; PMv, ventral premotor cortex. Reproduced from Errante et al. (2022).

*Structural connectivity* refers to the neural fibers pathways connecting distant brain areas, and can be approached using diffusion tension imaging (DTI) and diffusion spectrum imaging (DSI). A line of research analyzed the effects of brain disconnections on cognitive functions, such as language, to identify the underlying neural networks (Thiebaut de Schotten et al., 2020). These approaches can now be extended to the study of BSC. Pacella et al. (2019), for example, analyzed direct damage and disconnections from neuroimaging data in 174 patients with a right hemisphere stroke. They showed that three neural networks contribute significantly to anosognosia for hemiplegia, indicating that the premotor network, the limbic system and the ventral attentional network were involved in motor consciousness. To identify the brain network underpinning the sense of body ownership, Errante et al. (2022) analyzed structural brain connectivity in patients with stroke, who misidentified other individual’s limb as their own. The authors quantified the probability of disconnection of subcortical tracts in 70 patients with stroke presenting or not abnormal limb ownership and used DTI in a subsample of these patients. They found that altered sense of limb ownership was mainly related to disconnection of the arcuate fasciculus and the third branch of the superior longitudinal fasciculus (Figure 3). Thus, deficits in the sense of limb ownership resulted from the disconnection between frontal (ventral premotor cortex), parietal (intraparietal sulcus) and temporal (extrastriate body area) areas (Errante et al., 2022). Although ownership for a body part may be less relevant for BSC than global, whole-body ownership, the brain mechanisms involved appear to be similar (Ionta et al., 2014).

*Functional connectivity* is based on statistical dependence (e.g., measure of correlation or coherence) between signals recorded from distant brain areas. For example, functional connectivity can be obtained by calculating the correlation between BOLD signal fluctuations from distant brain regions during experimentally-induced changes in BSC (using full-body illusions modifying global body ownership and self-location). Ionta et al. (2014) investigated the network of areas functionally connected with the temporo-parietal junction, a core area encoding the sense of self-location and first-person perspective (Ionta et al., 2011b). The authors found that the temporo-parietal junction was bilaterally connected to the supplementary motor area, ventral premotor cortex, insula, intraparietal sulcus, and the occipito-temporal cortex. In addition, the authors reported that the strength of functional connectivity between the right temporo-parietal junction and the right insula reflected more strongly the perceived self-location and first-person perspective. These regions have consistently been related to global aspects (whole-body ownership and self-location) of BSC in previous neuroimaging studies [reviewed in Blanke (2012), Serino et al. (2013)].

Functional connectivity can also be assessed from stereo-electroencephalography (intracranial recordings) during presurgical evaluation of epilepsy, for example by calculating non-linear regressions between EEG signals recorded from distant intracranial electrodes (Bartolomei et al., 2017a,b). Popa et al. (2019) retrospectively analyzed the responses to electrical stimulation of the cingulate cortex in 110 patients with epilepsy. Twelve patients reported distorted BSC and body image during electrical stimulation of the cingulate cortex. Changes in BSC and body image were associated with a significant decrease in functional connectivity of the cingulate cortex with the left posterior insula (and to a lesser extent the left anterior insula), as well as with the prefrontal cortex, supplementary motor area, premotor cortex, primary somatosensory and motor cortices, and with the frontal and parietal opercula. In addition, changes in BSC were associated with increased functional connectivity between the anterior cingulate cortex and dorsolateral prefrontal cortex, as well as between temporal areas, the right anterior insula and supplementary motor area, or the right posterior insula.

## 3. Main challenges and perspectives

We focus here on three main challenges that may have hampered progresses in the understanding of the multisensory mechanisms and the neural bases of BSC: the lack of crosstalk and cross-fertilization between different subdisciplines and approaches from integrative neuroscience to decipher the neural bases of BSC; the often correlational rather than causal evidence that specific brain areas are instrumental in generating BSC; and the need for studies tapping into interindividual differences in the phenomenal experience of BSC and their underlying mechanisms. Some perspectives are summarized in Table 1.

**TABLE 1:** Some research perspectives.

∙ Improve cross-talk and cross-fertilization between levels of analysis of BSC
∙ Build conceptual bridges to better integrate multiple levels and methods of analyses of BSC
∙ Provide stronger causal evidence of involvement of neural networks in BSC
∙ Describe better the evolutionary aspects of BSC and its neural bases
∙ Describe the systems of neurotransmitters or hormones regulating BSC
∙ Analyze better interindividual differences in BSC and their neural underpinning, explaining variety in the experience of self from neurotypical to other populations
∙ Analyze overlap and differences between the neural bases of BSC and high-level aspects of the self
∙ Investigate further interrelations between the neural bases of BSC and the immune system

### 3.1. Lack of cross-talk and cross-fertilization between subdisciplines of integrative neuroscience to decipher the neural bases of BSC

**FIGURE 4:**
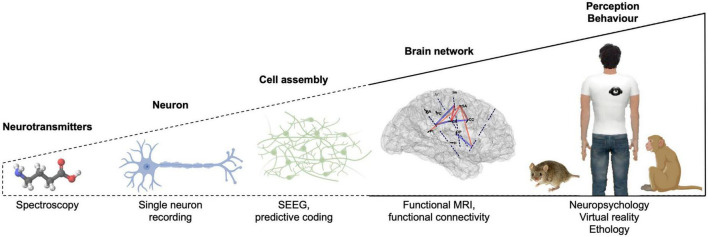
Levels of analysis within the field of integrative neuroscience to decipher the neural bases of bodily self-consciousness (BSC) and examples of techniques and approaches. Dashed lines indicate the levels of analysis that have been overlooked.

Integrative neuroscience is characterized by the investigation of brain functions with several levels of analysis, ranging from molecular (genes, neurotransmitters, receptors) and cell levels (action potentials) to cell assemblies, brain networks (whole-brain imaging) and cognition/behavior (ethology, neuropsychology), using a variety of methods (Figure 4). Multi-level analyses are required to understand complex behaviors, body-mind relations, or the neural bases of BSC. Each level of analysis depicted in Figure 4 can provide important insight into the neural bases of BSC. However, we note that there have been so far little interactions and cross-fertilization between the different levels of analyses within the field of integrative neuroscience, which have focused on the bodily self and their neural bases:

● *Molecular (genes, neurotransmitters, receptors) levels*. Studies into molecular levels are important to understand the biological bases of the neural tissue and its environment (structure and functions of the cells, synapses, receptors, and systems of neurotransmitters) that support BSC. This is important for clinical research as various microdeletions of genes may manifest with intellectual disability, personality disorders and altered sense of self. For example, there is an increased risk of schizophrenia in 22q11.2 deletion syndrome (Goldenberg et al., 2012), characterized by distorted sense of agency (i.e., erroneous attribution of actions to the self) (Salomon et al., 2022). We note that there have been to our knowledge only very few attempts to link BSC to neurochemistry and neuronal mechanisms, despite methods and techniques are available in animal and human research models. For example, Wada et al. (2019) used an original paradigm evoking illusory ownership over a fake body part in mice (the so-called rubber tail illusion). They investigated the intensity of the rubber tail illusion in *Caps2*-KO mice, that exhibit autistic-like phenotypes by reducing brain-derived neurotrophic factor (BDNF) release in the brain. They showed that *Caps2*-KO mice had lower rate of behavioral responses compatible with illusory ownership than the wild-type mice. This indicates that BDNF seems important for plastic events related to BSC in the parietal cortex, in line with observations in non-human primates and in humans (Ishibashi et al., 2002; Hiramoto et al., 2017). Despite this interesting line of research, only few studies directly investigated the systems of neurotransmitters or hormones related to BSC regulation. It would especially be important to demonstrate that the networks that have been linked to BSC are actually instrumental in the generation of BSC, just as the implication of the dopaminergic ventral tegmental area in consciousness (Spindler et al., 2021). Future studies should endeavor to analyze how different systems of neurotransmitters and neurons support BSC. Functional magnetic resonance spectroscopy, for example, would be a useful, non-invasive approach to provide *in vivo* quantification of brain regional biochemistry (Stanley and Raz, 2018).

● *Neuronal level.* Studies into the neurophysiological activity of neurons (action potentials) is especially important to investigate bodily experiences underpinning BSC as they can reveal the multisensory integration rules of signals from the body and its surrounding. Multisensory integration properties of neurons have been related to experimentally-induced changes in body part ownership or changes in the body schema (Iriki et al., 1996; Graziano et al., 2000). Although results from experimentally-induced changes in BSC in humans have been interpreted in the light of single cell recordings in non-human primates, there have been only little attempts to replicate these illusions in non-human primates, rodents, or other animal species.

● *Cell assembly and brain network level*. BSC and consciousness more generally are not reducible to the functioning of a few neurons or of a given brain area, but are rather seen as an emergent property of the brain, based on the communication of signals across large groups of neurons (cell assemblies) in distant brain regions (Godwin et al., 2015). Studying the synchrony between cell assemblies is deemed essential to understand how functionally and anatomically connected networks of neurons in the cortex and subcortical structures underpin certain behaviors/cognitive states (Oberto et al., 2022). Functional MRI, PET and EEG studies have described some neural *correlates* of BSC [reviewed in Blanke (2012), Serino et al. (2013)], but *causal evidence* of implications of certain cell assemblies in BSC need to be consolidated (see section “3.2 Paucity of causal evidence, instead of correlational evidence, in the study of the neural bases of BSC”), together with the description of their connectivity and pattern of communication (Uhlhaas et al., 2009). Just as for the analysis at the neuronal level, it will be important to compare the functioning of cell assemblies in human and non-human primates to study evolutionary aspects of BSC (Parvizi and Kastner, 2018).

● *Behavioral level: ontogenetic and phylogenetic approaches*. Studies into BSC at the behavioral level can be achieved using approaches from experimental psychology, psychophysics, cognitive neuroscience, or neuropsychology. Combining ontogenetic and phylogenetic approaches of BSC will be especially important to understand the emergence of the self (and its bodily and cognitive components) and self-consciousness. BSC has been investigated in infants (Cowie et al., 2018), in adults, and more rarely in adolescents and aging populations. However, there is to our knowledge no clear description of the patterns of development and changes in the neural underpinnings of BSC that occur throughout the life span. In addition, how developmental aspects of BSC relate to phylogenetic development of BSC has, to our knowledge, not been systematically investigated. Although there is evidence that even invertebrates show representation of the extent of their body (Sonoda et al., 2012), or that non-human primates can adopt the visuo-spatial perspective of other individuals (Karg et al., 2016), there is no clear answer as to how and to what extent BSC (and the neural structures at its basis) developed in vertebrates and invertebrates.

Although multi-level analyses are required to understand complex behaviors, theoretical articles have already highlighted the difficulty to integrate subdisciplines of neuroscience and to synthetize large data sets ranging from neurochemistry to whole-brain imaging (Kotchoubey et al., 2016). It has been pointed that “the usual solely additive combination of methods and levels may not be sufficient to construct a comprehensive picture of neuropsychological phenomena. Only theoretical efforts seem to promise integration by building conceptual bridges. Integration is not a juxtaposition of concepts, but rather their overlap” (Kotchoubey et al., 2016, p. 5; Buhusi et al., 2023). Thus, integrating data from different levels of analysis would be the true challenge for the neuroscientific investigations of BSC and its neural underpinning.

There are examples of successful multi-level approaches within the field of integrative neuroscience (e.g., neural underpinnings of working memory, locomotor behavior, motor control, and social behavior) (Grillner et al., 2005; Cacioppo and Decety, 2011; Kotchoubey et al., 2016; Buhusi et al., 2023). However, integrative approaches of BSC are rather rare and data must often be gathered from related but different research areas. For example, investigations into the neural bases of self-location (the experience of occupying a volume of space) can be linked to studies of the neural bases of spatial navigation and spatial memory, which have been deciphered in several animal models (e.g., Ulanovsky and Moss, 2007; Killian et al., 2012; Cullen and Taube, 2017; Grieves and Jeffery, 2017; Moser et al., 2017), or in humans using intracranial EEG recordings (e.g., Ekstrom et al., 2003). Similarly, understanding the neural bases of the sense of agency – approached to date mostly through correlative studies [e.g., Farrer and Frith, 2002 reviewed in Charalampaki et al. (2022), Abdulkarim et al. (2023)] and a few causal studies (Dary et al., 2023) may require to compile results from studies on motor control, volition, intentional movement and sensory feedback (Haggard, 2017). There is indeed only rare studies in non-human primates or other animal models that directly investigated the sense of agency (Kaneko and Tomonaga, 2011; Couchman, 2015).

We suggest that the integrative approach was particularly successful for the investigation of the experience of body ownership, as an effort has been made to link various levels, methodologies, and fields, including philosophy (de Vignemont, 2011; Blanke, 2012; de Vignemont and Alsmith, 2017). As noted above, the rubber hand illusion (Botvinick and Cohen, 1998; Tsakiris and Haggard, 2005) has been extensively used as an experimental paradigm to understand the phenomenal and multisensory levels of limb ownership, as well as their neural bases. At a behavioral level, illusory ownership over a fake hand or avatar has been investigated in healthy humans and in various clinical populations. However, only few studies adapted this paradigm to other animal species, including monkeys (Graziano et al., 2000; Fang et al., 2019) and more recently mice (Wada et al., 2016, 2019; Buckmaster et al., 2020), also showing that synchronous visuo-tactile stroking on a body part placed in an anatomically plausible posture can elicit illusory ownership. The paradigm has also been adapted to fMRI, EEG, PET, intracranial EEG, allowing to understand how very large neuronal assemblies contribute to BSC. With the exception of single cell recordings in parietal area 5 in monkeys, showing that response of bimodal visuo-proprioceptive neurons change when a monkey looks at a taxidermized arm being stroked in synchrony with its own arm (Graziano et al., 2000), we do not know to what extend this applies to other brain areas and how is the sense of owning a body part coded at single neuron level and small neurons assemblies. Interestingly, there have been a few pharmacological studies that have shown increased rubber hand illusion intensity in individuals taking ketamine (Morgan et al., 2011) or an interaction between the sense of body part ownership and the immune system (Finotti and Costantini, 2016; Finotti et al., 2018). Similar multilevel approaches should now be extended to all bodily experiences underlying BSC, with a stronger emphasis on phylogenetic aspects of BSC (interspecies), and interindividual differences in BSC (Table 1).

### 3.2. Paucity of causal evidence, instead of correlational evidence, in the study of the neural bases of BSC

Whereas fMRI and PET are considered to show neural *correlates* of BSC, a *causal* demonstration of the involvement of specific brain regions in BSC can be provided by non-invasive and invasive direct brain stimulation (Siddiqi et al., 2022; Elmalem et al., 2023).

*Non-invasive brain stimulation* modulates the excitability of the brain using transcranial magnetic stimulation (TMS) and transcranial direct-current stimulation. For example, van Elk et al. (2017) used transcranial direct-current stimulation to investigate the role of the right temporo-parietal junction in perspective taking. The authors found that anodal stimulation of the right temporo-parietal junction reduced the performance when participants mentally simulated being in someone else’s shoes (i.e., third-person perspective taking). Similarly, Martin et al. (2020) found that anodal stimulation to the right temporo-parietal junction increased the effect of the participant’s body position (congruent or not with that of a seen avatar) in a third-person perspective taking task. These studies stressed that the right temporo-parietal junction is causally involved in perspective tacking.

A recent TMS study focused on real-time brain activity underlying the sense of body ownership (Casula et al., 2022). The authors coupled TMS over the hand region in the primary motor cortex with EEG recordings in 19 healthy participants immersed in a virtual environment in which virtual limbs were displayed. Casula et al. (2022) showed a decrease in cortical activity in the hand region of the primary motor cortex contralateral to the virtual hand participants self-identified with. Moreover, this study showed that illusory hand ownership was related to increased connectivity with the posterior parietal cortex, and decreased connectivity with the premotor cortex, indicating plasticity in the fronto-parietal networks associated with the ownership component of BSC.

*Invasive brain stimulation* is used to map brain functions during presurgical evaluation of focal intractable epilepsy (Ritaccio et al., 2018; Mercier et al., 2022) and in awake patients during brain tumor resection (Duffau, 2015). During electrical brain stimulation, a current of several mA is delivered to the brain through implanted electrodes or through subdural grids of electrodes placed at the surface of the cerebral cortex. Recently, several studies have reported the effects of direct electrical brain stimulation on BSC. For example, patients stimulated in the precentral gyrus, superior frontal gyrus, or in the planum temporale, reported disturbance of agency, while stimulation in the inferior temporal gyrus, middle occipital gyrus and middle frontal gyrus evoked changes in self-location, such as a feeling to “get out of his/her body” (Andelman-Gur et al., 2020). A systematic review of the literature (Dary et al., 2023) showed that only electrical stimulation of the parietal cortex disturbed all core components of BSC considered in the review article (i.e., body ownership, self-location, agency, first-person perspective) and the proportion of responses evoked by electrical stimulation in the parietal cortex was higher than in any other brain area stimulated. Except for disturbance of body ownership, which was only evoked once by stimulation of the superior parietal lobule, stimulation of the inferior parietal lobule altered all other core components of BSC. The inferior parietal lobule (angular gyrus and supramarginal gyrus) was also the area where the spatial density of electrical brain stimulation distorting the bodily self showed the strongest overlap (Figure 2C). However, this retrospective analysis of published cases lacked structured questionnaires or interview that are required to describe the complex experience associated with changes in the bodily self. In addition, this analysis included only few studies providing whole-brain mapping, with rare studies of the subcortical structures and no study of the brainstem. Prospective studies should now provide whole-brain mapping (cortical and subcortical) of the structures causally involved in BSC by describing effects of direct electrical brain stimulation in large cohorts of patients with epilepsy or brain tumors, using novel methods to identify the focal and connective organization of brain networks (see Elmalem et al., 2023).

### 3.3. Considering interindividual differences in BSC and its neural mechanisms

Although the rubber hand illusion and the full-body illusion have been showed to modify BSC at the group level, studies revealed interindividual differences in the vividness of these illusions. One explanation given to these interindividual differences focused on the role of empathy (Nakul et al., 2020b), suggesting that participants with higher empathy scores were more prone to experiencing the illusion. Accordingly, individuals with autism spectrum disorders, who are less empathic, were less sensitive to body illusions than control participants (Mul et al., 2019). In addition to personality traits such as empathy, another explanation takes into account individual differences in how the brain weights tactile, visual, vestibular, interoceptive and proprioceptive information for interpreting bodily experiences underling BSC (Pfeiffer et al., 2013; David et al., 2014). Individuals trained to use proprioceptive and interoceptive signals, such as professional dancers (Ehrsson, 2012; Virtanen et al., 2022), might be less prone to different types of bodily illusion (rubber hand illusion, full-body illusion).

Other studies tried to explain interindividual differences by variations in the cortical thickness in areas underpinning BSC. Kanayama et al. (2017) investigated the relationship between subjective reports about agency, ownership, or the narrative self, and gray matter volume in 96 healthy participants. They found a significant correlation between gray matter volume in the insula and subjective reports. The ownership score correlated with gray matter volume in the postcentral gyrus, insula and angular gyrus. Another study investigated whether cortical thickness correlated with interindividual differences in illusory body ownership during the rubber hand illusion (Matuz-Budai et al., 2022). Results from this study showed that subjective reports correlated positively with cortical thickness in several areas, such as the insula, precuneus, postcentral gyrus, lateral occipital cortex, middle temporal gyrus and superior temporal gyrus. Altogether, these results indicate that the morphology of some brain structures might explain in part the interindividual differences in the bodily illusion and BSC.

The different factors explaining the interindividual differences in the experience of being a self, including the morphology of the brain, the different patterns of functional and structural connectivity, or the functioning of the metabolic, immune and neurochemistry systems, should be taken into account more systematically in future studies.

## 4. Conclusion

The investigation of the neural bases of BSC has been a very active and productive field of research during the last 10 years. These studies have extended our understanding of the multisensory contributions to the neural underpinnings of BSC, reinforcing for example the role of interoceptive signals in BSC (Park and Blanke, 2019). Recent studies have also endeavored to identify the commonalities and differences between the brain networks underpinning the pre-reflective and immediate bodily self from higher-level forms of “cognitive” self. However, we noted a lack of cross-talk and cross-fertilization between levels of analyses, as well as the paucity of data regarding the molecular and cellular mechanisms underlying the neural bases of BSC (Table 1). This lack of data not only limits the interpretation of studies investigating phenomenal aspects of BSC, but also hampers the understanding of the mechanisms leading to abnormal forms of BSC in neurodevelopmental and psychiatric disorders (Tordjman et al., 2019). A better understanding of the neural bases of BSC will also be especially important for a better assessment of patients with brain lesions, or to understand and predict more accurately the consequences of brain surgery (e.g., resection of glioma or epileptogenic zone) on the various components of BSC (Schaller et al., 2021).

## Author contributions

ZD and CL drafted, corrected, and finalized the manuscript. Both authors contributed to the article and approved the submitted version.

## Footnotes

1
https://www.frontiersin.org/research-topics/43343/insights-in-integrative-neuroscience-2022#overview
